# Association Between Partial Pressure of Carbon Dioxide and Immediate Seizures in Patients With Primary Intracerebral Hemorrhage: A Propensity-Matched Analysis

**DOI:** 10.3389/fneur.2022.865207

**Published:** 2022-04-21

**Authors:** Zhiming Pan, Qiuli Zhong, Chaoying Wang, Jianqun Wang, Xiaoyan Chen, Xiaoyan Li, Xintong Zhang, Yibin Zhang

**Affiliations:** ^1^Department of Neurosurgery, Dehua County Hospital, Quanzhou, China; ^2^Department of Internal Medicine, Dehua County Hospital, Quanzhou, China; ^3^Department of Neurosurgery, Yuebei People's Hospital, Shaoguan, China; ^4^Department of Neurosurgery, Neurosurgery Research Institute, The First Affiliated Hospital, Fujian Medical University, Fuzhou, China

**Keywords:** intracerebral hemorrhage, carbon dioxide, epilepsy, risk factor, stroke

## Abstract

**Purpose:**

To explore the value of partial pressure of carbon dioxide (PaCO_2_) levels in arterial blood for predicting immediate seizures (ISs) in patients with primary intracerebral hemorrhage (ICH).

**Methods:**

Demographic information and clinical data from patients with primary ICH were prospectively collected, including arterial blood gas analysis. Immediate seizures (ISs) were determined as seizures in the first 24 h after admission. Univariate and multivariate analyses were performed to assess the association of PaCO_2_ levels with ISs. Propensity-score matching (PSM) analyses were adopted to reduce the baseline difference between ISs and non-ISs groups.

**Results:**

A total of 596 patients with primary ICH were initially screened in this clinical study, 368 of whom fulfilled all the inclusion criteria [mean age, (60.46 ±12.78) years; 57.9% female patients]. ISs occurred in 30 of the 368 (8.15%) patients with primary ICH of this cohort. Patients with ISs had significantly lower PaCO_2_ levels [34.35(32.38–37.53) vs. 39.45(35.90–43.43), mmHg, *p* < 0.001] and were younger than those without ISs [(54.57±12.15 vs. 60.99 ±12.72) years, *p* = 0.008]. Multivariate analysis showed that lower initial PaCO_2_ (≤37.2 mmHg) level was a significant independent predictor of ISs [odds ratios (OR) 0.141, 95% confidence interval (CI) 0.057–0.351, *p* < 0.001], as well as younger age (OR 0.961, 95% CI 0.928–0.995, *p* = 0.023) and hematoma expansion (OR 0.340, 95% CI 0.134–0.863, *p* = 0.023). Receiver operating characteristic curve (ROC) analysis demonstrated that the optimal cutoff value of PaCO_2_ level for predicting ISs was 37.20 mmHg in patients with primary ICH (the area under the curve (AUC) was 0.760 with a corresponding sensitivity of 76.67% and specificity of 67.46%, 95%CI = 0.713–0.802, *p* < 0.001). After PSM, the matched ISs group had significantly lower PaCO_2_ levels compared with the matched non-ISs group [34.45(32.43–38.18) vs. 41.75(35.85–43.98) mmHg, *p* < *0*.05] in the univariate analysis. The lower initial PaCO_2_ level was still independent of ISs following primary ICH.

**Conclusions:**

The lower initial PaCO_2_ level was associated with an increased risk of ISs in patients with primary ICH.

## Introduction

Intracerebral hemorrhage (ICH) occurs in 15–25% of all strokes and is the most devastating and untreatable type of hemorrhagic stroke with a high risk of disability and mortality (1, 2). Seizures are a frequent and intractable complication of ICH, and they vary widely among epidemiological studies, ranging from 2.5 to 28% (3–9), with the majority occurring at or near onset (10, 11). The reported literature generally classifies seizures by the time of onset as immediate (<24 h after admission), early (1–14 days), or late (>2 weeks) (12). Most animal models have shown that the first seizure occurred in the first 24 h post-ICH (13). Clinically, ~90% of the seizures occur within the first 3 days post-ICH (11, 14, 15). Following ICH, the risk factors for immediate seizures (ISs) include young age and lobar location of ICH (5, 12).

Carbon dioxide (CO_2_) plays a crucial role in neuronal metabolic activity, neurotransmitter function, and regulating cerebral blood flow (CBF) (16, 17). Acute hemorrhagic stroke, including ICH, appears to correlate with a significant decrease in CO_2_ levels (10). Published animal studies have demonstrated that CO_2_ levels influence tissue pH, which plays a vital role in developing acute epilepsy (18, 19). Previous literature indicated that CO_2_ concentrations and respiratory mechanisms might be associated with seizures (20–22). The hippocampus and cerebral cortex, frequently involved in seizures, are susceptible to CO_2_ and may mediate a neuroendocrine response to CO_2_ (19). Prior studies demonstrated that lower partial pressure of carbon dioxide (PaCO_2_) was associated with febrile convulsions or the absence of seizures in children (23, 24). One recent study revealed that early decreased PaCO_2_ levels in the first 24 h were independently associated with an increased incidence of acute seizures in patients receiving extracorporeal membrane oxygenation for respiratory failure (25). To date, the association of PaCO_2_ with ISs following ICH has not previously been reported in the literature (12, 26). Also, Hextrum et al. have reported that the initial PaCO_2_ level is a stronger predictor of disease progression than the 72-h nadir (27). We, therefore, sought to test the hypothesis whether the initial PaCO_2_ level was associated with ISs in patients with primary ICH.

## Materials and Methods

### Study Population

**Figure 1:**
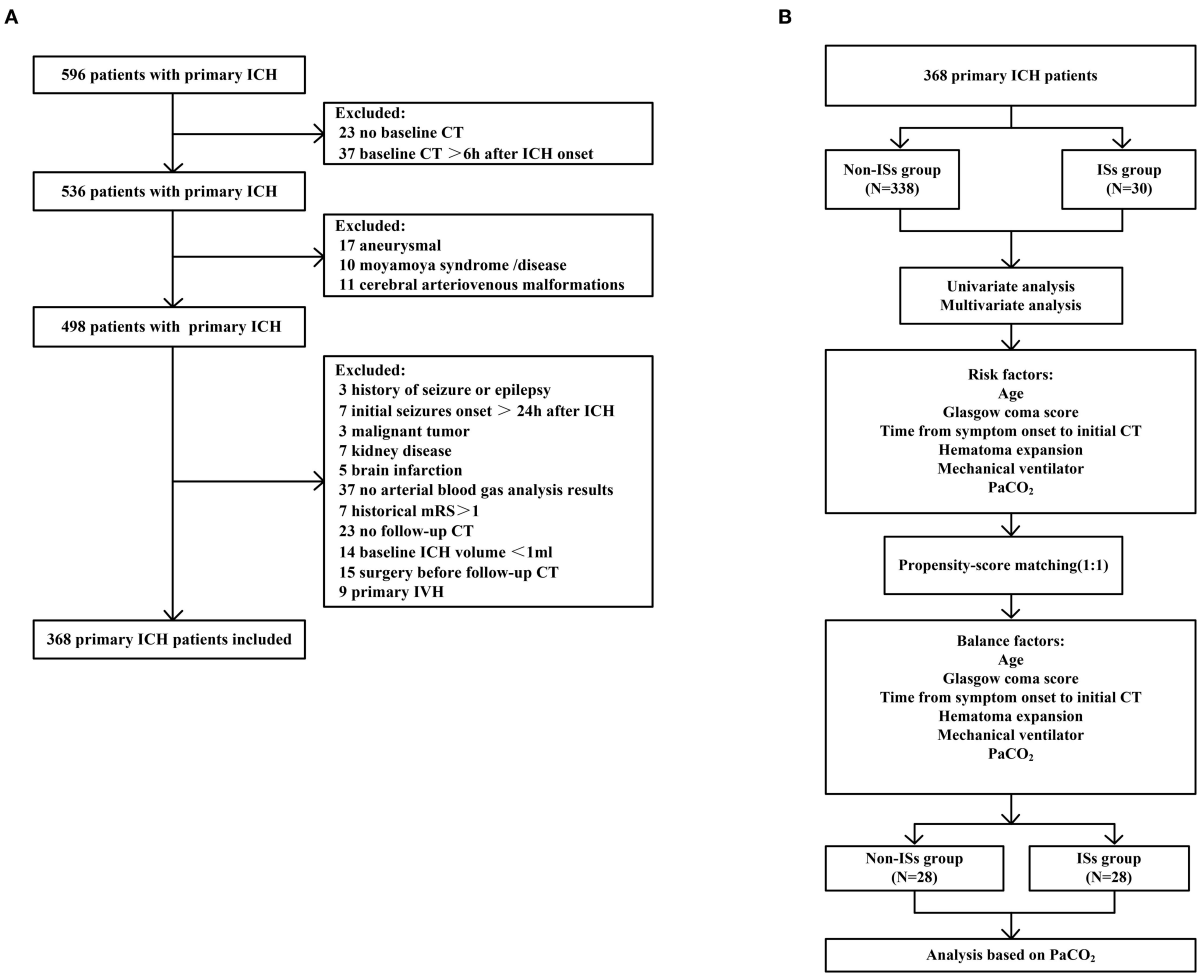
Flow diagram of the patient selection process. **(A)** Flowchart of patient inclusion; **(B)** Flowchart of propensity-score matching. ICH, intracerebral hemorrhage; IVH, intraventricular hemorrhage.

Patients with ICH presenting directly to Dehua County Hospital from January 7 2018 to 21 May 2021, were enrolled in the study. Immediate seizures (ISs) were determined as seizures in the first 24 h after admission (5, 12, 28). Neurosurgeons or nurses witnessed ISs in the hospital. The inclusion criteria were: (1) baseline computed tomography (CT) within 6 h after hemorrhage was performed in all patients, and a follow-up CT scan was performed within 24 h; (2) computerized tomography angiography (CTA) was completed within 72 h of admission to exclude cerebrovascular diseases such as intracranial aneurysms, cerebral arteriovenous malformations, and moyamoya disease; (3) arterial blood gas analysis was performed within 2 h of admission. The exclusion criteria were as follows: (1) patients underwent emergency surgery before follow-up CT;(2) patients with suspicious and pre-admission seizures described by family members; (3) patients with hemorrhage induced by brain infarction, vascular malformations, and a brain tumor; (4) an initial ICH volume <1 ml; (5) primary intraventricular hemorrhage (IVH); (6) acute kidney injury or chronic kidney disease; (7) historical modified Rankin Scale (mRS) scores >1; (8) malignant tumor. Study inclusion/exclusion is summarized in Figure 1.

Antiepileptic drugs (AEDs), including sodium valproate, phenobarbital, or levetiracetam, were given if clinical seizures were observed. Multiple AEDs were used in combination when seizures were poorly controlled.

### Data Collection

Demographic information and clinical data, including age, sex, medical history, vital admission signs, baseline Glasgow coma score (GCS) score, time from symptom onset to initial CT, baseline ICH volume, primary IVH, ICH location, arterial blood gas analysis, admission laboratory, hematoma expansion (HE), treatment received, mechanical ventilator, and all other data related to their hospitalization were prospectively collected. Arterial blood gas analysis, including PaCO_2_, was conducted within 2 h after arrival at our department and simultaneously obtained using i-STAT Analyzer (Abbott Park MN: 300-G, Singapore).

### CT Scan Analysis

Patients with primary ICH were categorized into two groups for the statistical analysis: patients with deep or lobar ICH. Primary ICH with selective involvement of the thalamus, basal ganglia, internal capsule, deep periventricular white matter, or brain stem was defined as deep ICH, whereas ICH isolated in the cortex (with or without subcortical white matter involvement) was defined as lobar ICH (29). Follow-up CT was carried out within 24 h after hospital admission to identify a HE diagnosis. ICH volumes were calculated using a post-processing workstation (Advantage Workstation 4.6, GE Healthcare, Chicago, Illinois, USA) (30). HE was defined as a 33% increase in the hematoma volume or >6 mL on follow-up imaging (31).

### Clinical Prognostic Assessment

Functional outcome was measured at 90 days after ICH onset with the modified Rankin Scale (mRS) score. A favorable outcome was defined as an mRS score of 0–2, and an unfavorable outcome was defined as an mRS score of 4 or greater (32).

### Statistical Analysis

Normally distributed continuous variables were presented as mean ± standard deviation (SD), non-normally distributed continuous variables as median (interquartile range, IQR), and categorical variables as frequencies (percentages). Median with IQR was shown for violin plots. Categorical data were analyzed by chi-squared test or Fisher's exact test. Normally and non-normally distributed variables were analyzed for significance by Student's t-test or Mann–Whitney U test, respectively. The correlation was determined using the Spearman rank test. Risk factors with *p* < 0.1 in univariate analyses were included in the multivariate models. Moreover, the receiver operating characteristic (ROC) curve was plotted by MedCalc (MedCalc Software, Ostend, Belgium), and the area under the ROC curve (AUC) was calculated to evaluate the predictive power of PaCO_2_ for ISs. The best cutoff value of PaCO_2_ for predicting ISs was selected based on the ROC cutoff value, and patients were divided into “ ≤ optimal cutoff value” and “>optimal cutoff value.” Propensity score matching (PSM) analysis was implemented with a 1:1 nearest-neighbor matching algorithm to balance confounders between the two groups. Variables with *p* < 0.05 in the univariate analysis were entered into the PSM. All statistical analyses were performed using SPSS Statistics 25.0 software (SPSS Inc., Chicago, USA) and Prism 8.3.0 (GraphPad Software, San Diego, CA, USA). Differences were considered statistically significant when *p* < *0.05*.

## Results

**Figure 2:**
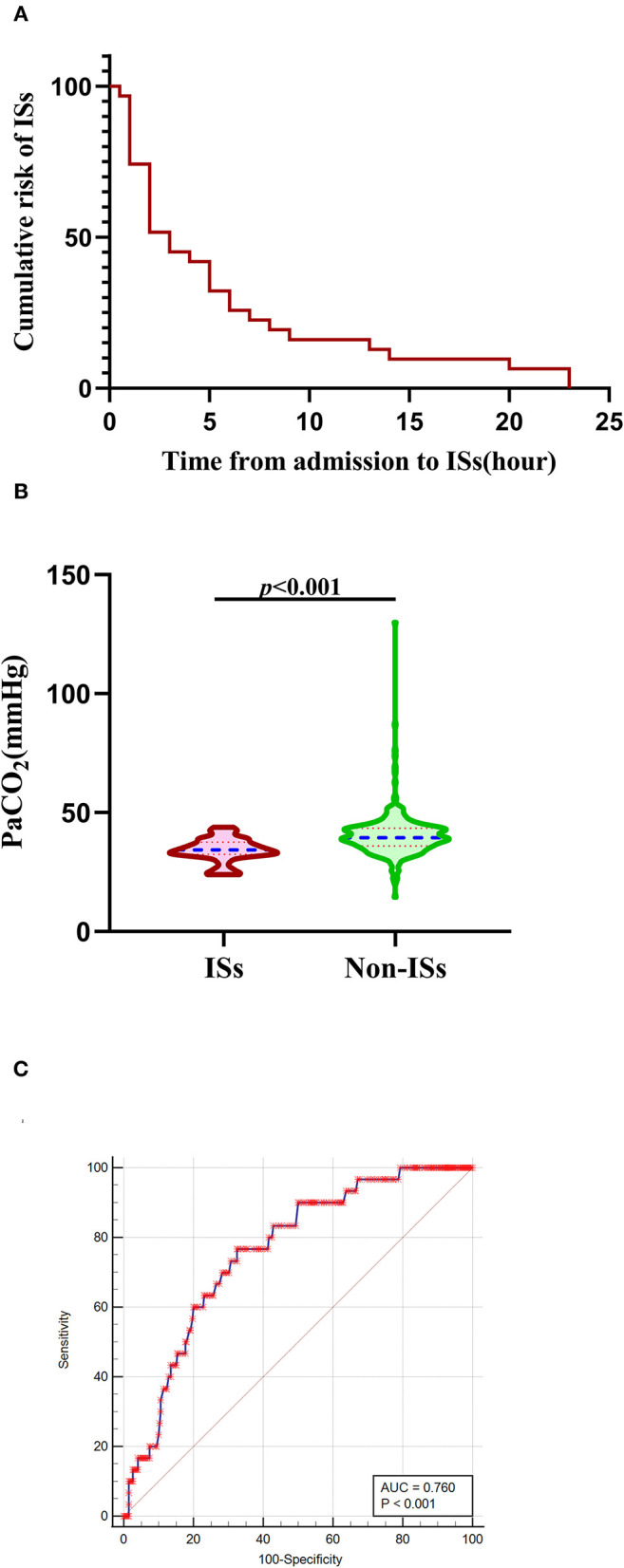
Association of initial PaCO_2_ levels with ISs. **(A)** The cumulative risk of ISs using Kaplan-Meier analysis within 24 h after admission in the ISs group. (B) PaCO_2_ levels in patients with ISs (*n* = 30) and non-ISs (*n* = 338). **(C)** ROC curve analysis for predicting ISs. The optimal cutoff value of PaCO_2_ level for predicting ISs was 37.20 mmHg in patients with primary ICH (the AUC was 0.760 with a corresponding sensitivity of 76.67% and specificity of 67.46%, 95%CI = 0.713–0.802, *p* < 0.001). Median with IQR was shown for all violin plots in panel B. Mann–Whitney tests were performed to compare differences between groups. AUC, area under the curve; ICH, intracerebral hemorrhage; ISs, immediate seizures; IQR, interquartile range; PaCO_2_, partial pressure of carbon dioxide; ROC, Receiver operating curve.

As described in Figure 1 (the detailed flow of the selection process), a total of 596 patients with primary ICH were initially screened in this clinical study, 368 of whom fulfilled all the inclusion criteria [mean age (60.46 ±12.78) years; 57.9% female patients]. Thirty patients (8.15%) experienced ISs, and 53 patients developed early HE after primary ICH. The median baseline GCS score was 12.0 (7.0–15.0), and the median baseline ICH volume was 12.0 ml (5.0–27.00 ml). The median time from symptom onset to initial CT was 2 h (1–3 h), and the median time from admission to ISs onset was 3 h (1–7 h). Cumulative ISs rate using Kaplan–Meier analysis within 24 h after admission in the ISs group is illustrated in Figure 2A.

Baseline demographic and clinical characteristics are summarized in Table 1. The 368 patients with primary ICH were stratified into ISs-group (*N* = 30) and non-ISs group (*N* = 338). In the univariate analysis, significant differences were observed in age, mechanical ventilator (MV) within 24 h, GCS score, HE, and PaCO_2_ between the ISs and non-ISs groups (*p* < 0.05). The PaCO_2_ level was significantly lower in the ISs group than that in the non-ISs group [34.35 (32.38–37.53) vs. 39.45(35.90–43.43), mmHg, *p* < 0.001; Table 1 and Figure 2B]. Patients were younger in the ISs group than the non-ISs group (54.57±12.15 vs. 60.99 ±12.72, years, *p* = 0.008; Table 1). Multicollinearity analyses were carried out among the predictors included in the multivariate analysis model. A variable inflation factor (VIF) > 5 or tolerance <0.2 indicates the existence of multiple collinearity (33). We observed no significant collinearity between the covariates included in the multivariable models as judged by VIF and tolerance (Table 2). Therefore, six covariates were included in stepwise multivariate analysis. In the multivariate logistic regression model, lower initial PaCO_2_ (≤37.2 mm Hg) level was a significant independent predictor of ISs (OR 0.141, 95% CI 0.057–0.351, *p* < 0.001), as well as younger age [odds ratios (OR) 0.961, 95% confidence interval (CI) 0.928–0.995, *p* = 0.023; Table 3)] and HE (OR 0.340, 95% CI 0.134–0.863, *p* = 0.023; Table 3). However, baseline GCS score and MV within 24 h were not independent predictors of ISs (*p* > 0.05; Table 3)_._ The Hosmer–Lemeshow test, used to access good-of-fit for multivariate models, indicated that the model fitted to a satisfactory extent (χ^2^=5.896, *p* = 0.659). Interestingly, Spearman correlation analysis detected a positive correlation of PaCO_2_ with GCS score (*r* = 0.1055, *p* = 0.0432). ROC analysis for assessing the ability of initial PaCO_2_ to identify ISs is shown in Figure 2C. The optimal cutoff value of the initial PaCO_2_ level for predicting ISs was 37.20 mmHg in patients with primary ICH [the area under the curve (AUC) was 0.760 with a corresponding sensitivity of 76.67% and specificity of 67.46%, 95%CI=0.713-0.802, *p* < 0.001; Figure 2C].

**Table 1:** Univariate analysis of association with ISs before and after propensity-score matching in spontaneous intracerebral hemorrhage patients.

**Characteristics**	**Before propensity-score matching**			**After propensity-score matching**		
	**Non-ISs**	**ISs**	***P*-value**	**Non-ISs**	**ISs**	***P*-value**
	**(*N =* 338)**	**(*N =* 30)**		**(*N =* 28)**	**(*N =* 28)**	
Age, yrs, mean ± SD	60.99 ±12.72	54.57 ± 12.15	0.008	55.61 ± 10.71	54.71 ± 12.56	0.776
**Gender (** ***N*** **, %)**			0.161			0.397
Male	192(74.3)	21(70.0)		17(60.7)	20(71.4)	
Female	146(25.7)	9(30.0)		11(39.3)	8(28.6)	
**Admission vital signs**						
Temperature, °C, mean ± SD	36.49 ± 0.45	36.63 ± 0.42	0.116	36.59 ± 0.53	36.65 ± 0.42	0.635
SBP, mmHg, mean ± SD	184.74 ± 30.59	184.50 ± 40.76	0.969	190.25 ± 36.89	182.18 ± 40.48	0.439
DBP, mmHg mean ± SD	104.99 ± 18.39	100.70 ± 20.15	0.268	109.0 ± 22.52	100.43 ± 20.83	0.145
**Medical history**						
Hypertension (*N*, %)	314(92.9)3	26(86.7)	0.217	226(92.9)3	24(85.7)3	0.388388
Chronic obstructive pulmonary disease	100(29.6)	5(16.7)	0.133	6(21.4)	5(17.9)	0.737
Diabetes (*N*, %)	31(9.2)	3(10.0)	0.881	4(14.3)	3(10.7)	0.686
Coronary heart disease (*N*, %)	26(7.7)	3(10.0)	0.653	1(3.6)	3(10.7)	0.299
Baseline Glasgow coma score, median (IQR)	12(7.75–15.0)	9.50(5.75–12.50)	0.008	6.0(5.0–11.75)	9.50(5.25–13.50)	0.199
Time from symptom onset to initial CT, hours, median (IQR)	2.0(1.0–3.0)	1.0(1.0–2.0)	0.079	1.5(1.0–2.0)	1.0(1.0–2.0)	0.930
Baseline volume, ml, median (IQR)	12(5.0–26.27)	11.0(5.75–33.95)	0.495	10.03(6.25–23.95)	10.05(5.25–29.66)	0.954
**ICH location (N, %)**			0.855			0.783
Lobar	141(41.7)	12(40.0)		11(39.3)	10(35.7)	
Deep	197(58.3)	18(60.0)		17(60.7)	18(64.3)	0.078
Intraventricular hemorrhage (*N*, %)	64(18.9)	5(16.7)	0.760	5(17.9)	4(14.3)	0.716
MV within 24 h (*N*, %)	87(25.7)	16(53.4)	0.001	16(57.1)	14(50.0)	0.592
Hematoma expansion (*N*, %)	43(12.7)	10(33.3)	0.002	5(17.9)	8(28.6)	0.342
**Treatment**			0.289			0.365
Conservative therapy (*N*, %)	255(75.4)	20(66.7)		22(78.6)	19(67.9)	
Surgery (*N*, %)	83(24.6)	10(33.3)		6(21.4)	9(32.1)	
**Admission laboratory**						
Hemoglobin, g/L, mean ± SD	136.09 ± 19.89	138.80 ± 21.66	0.479	137.54 ± 20.70	140.54 ± 21.01	0.593
Hematocrit, %, mean ± SD	40.21 ± 5.35	40.72 ± 6.25	0.627	40.70 ± 5.75	41.19 ± 6.14	0.761
**Arterial blood gas analysis**						
Partial pressure of carbon dioxide, mmHg, median (IQR)	39.45(35.90–43.43)	34.55(32.38–37.53)	<0.001	41.75(35.85–43.98)	34.45(32.43–38.18)	0.001
Partial pressure of oxygen, mmHg, median (IQR)	96(76–125)	114(78.25–135.25)	0.231	91.50(77–112)	114(80–134.75)	0.174
Pondus hydrogenii, median (IQR)	7.40(7.37–7.43)	7.41(7.36–7.46)	0.333	7.39(7.36–7.41)	7.41(7.35–7.46)	0.232
Arterial oxygen saturation, median (IQR)	97(96–99)	97(95–99)	0.883	97(96–98.75)	97.5(95.0–99.0)	0.947
**modified Rankin Scale (** ***N*** **, %)**			0.001			0.515
0–2	163(48.2)	5(16.7)		7(25.0)	5(17.9)	
3–6	175(51.8)	25(83.3)		21(75.0)	23(82.1)	

*DBP, diastolic blood pressure; ISs, immediate seizures; IQR, Interquartile range; MV, mechanical ventilator; SBP, Systolic Blood Pressure*.

**Table 2:** Multicollinearity test for the factors of a multivariate model.

	**Multicollinearity statistics**	
**Independent Variable**	**Tolerance**	**Variable inflation factor**
Age	0.967	1.034
Baseline Glasgow coma score	0.523	1.913
Time from symptom onset to initial CT, hours, median (IQR)	0.931	1.074
Partial pressure of carbon dioxide ≤ 37.2 mmHg	0.963	1.039
Hematoma expansion	0.972	1.028
Mechanical ventilator within 24 h	0.533	1.877

**Table 3:** Predictors for ISs of spontaneous intracerebral hemorrhage in multivariate model.

	**Unadjusted**				**Adjusted**			
	**OR (95%CI)**				**AOR (95%CI)**			
**Independent variable**	**OR**	**Lower**	**Upper**	***P*-value**	**OR**	**Lower**	**Upper**	***P*-value**
Age	0.957	0.926	0.990	0.010	0.961	0.928	0.995	0.023
Baseline Glasgow coma score	0.891	0.813	0.977	0.014	0.976	0.850	1.120	0.726
Time from symptom onset to initial CT, hours, median (IQR)	0.944	0.842	1.059	0.326	0.962	0.863	1.072	0.480
Partial pressure of carbon dioxide ≤37.2mmHg	0.872	0.819	0.929	<0.001	0.141	0.057	0.351	<0.001
Hematoma expansion	0.292	0.128	0.664	0.003	0.340	0.134	0.863	0.023
Mechanical ventilator within 24 h	0.303	0.142	0.647	0.002	0.409	0.138	1.210	0.106

*CI, Confidence interval; OR, Odds ratios*.

**Figure 3:**
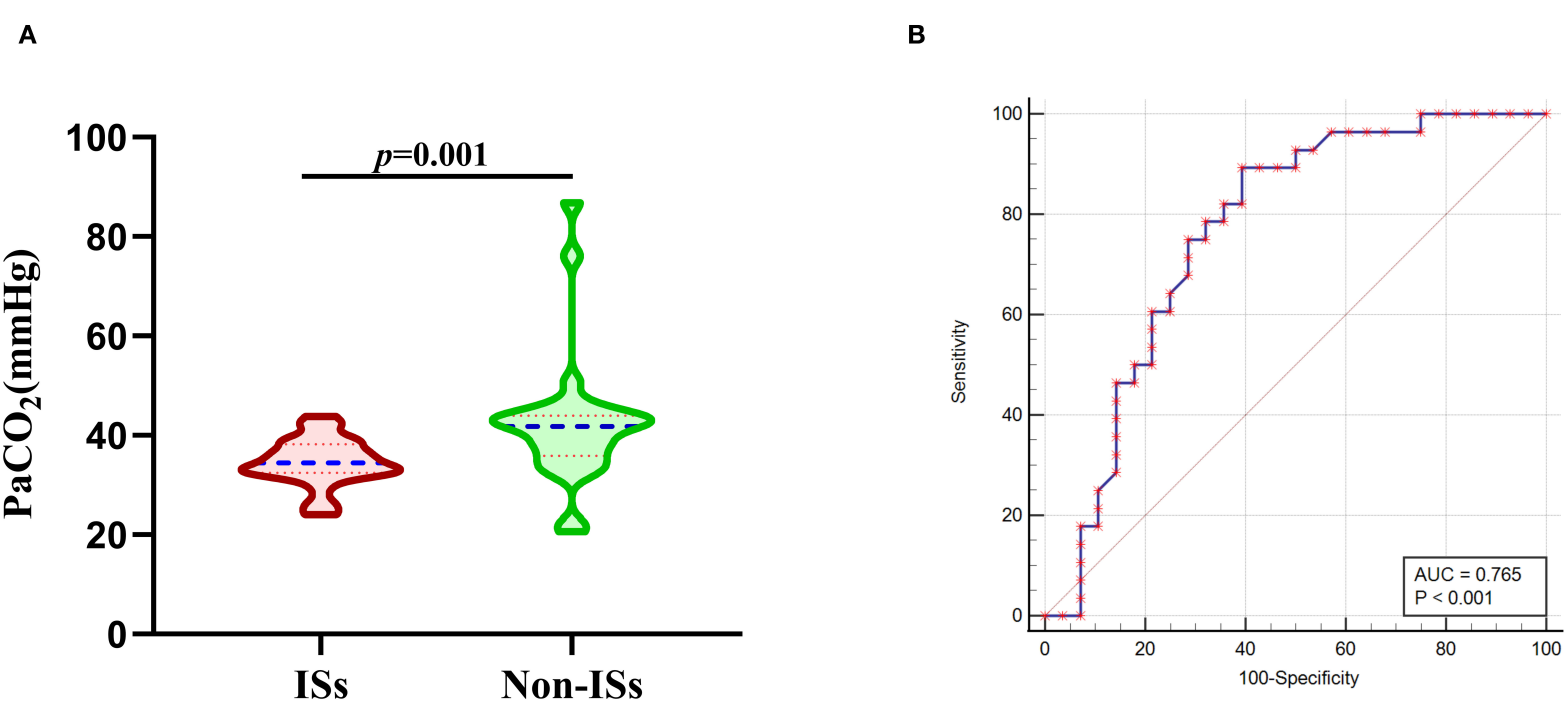
Association of initial PaCO_2_ levels with ISs after PSM. **(A)** PaCO_2_ levels in patients with ISs (*n* = 28) and non-ISs (*n* = 28) after propensity-score matching. **(B)** ROC curve analysis for predicting ISs after PSM. The optimal cutoff value for PaCO_2_ levels as a predictor for ISs following primary ICH was determined as 39.4mmHg (the AUC was 0.765 with a corresponding sensitivity of 89.29% and specificity of 60.71%, 95%CI = 0.632–0.868). Median with IQR was shown for all violin plots in **(A)**. Mann-Whitney tests were performed to compare differences between groups. AUC, area under the curve; ICH, intracerebral hemorrhage; ISs, immediate seizures; IQR, interquartile range; ISs, immediate seizures; PaCO_2_, partial pressure of carbon dioxide; ROC, Receiver operating curve.

To further account for significant differences in baseline characteristics between the ISs group and non-ISs group, we conducted a PSM. After PSM, two patients in the ISs group could not be matched. The significant differences in age, MV within 24 h, GCS score, and HE between the two groups were balanced. The matched ISs group had significantly lower PaCO_2_ levels compared with the matched non-ISs group [34.45(32.43–38.18) vs. 41.75(35.85–43.98) mmHg, *p* < *0*.05; Table 1 and Figure 3A)] in the univariate analysis. The lower initial PaCO_2_ level was still an independent predictor of ISs. After PSM, the optimal cutoff value for initial PaCO_2_ levels as a predictor for ISs following primary ICH was determined as 39.4 mmHg (the AUC was 0.765 with a corresponding sensitivity of 89.29% and specificity of 60.71%, 95%CI = 0.632–0.868, *p* < 0.001; Figure 3B).

**Figure 4:**
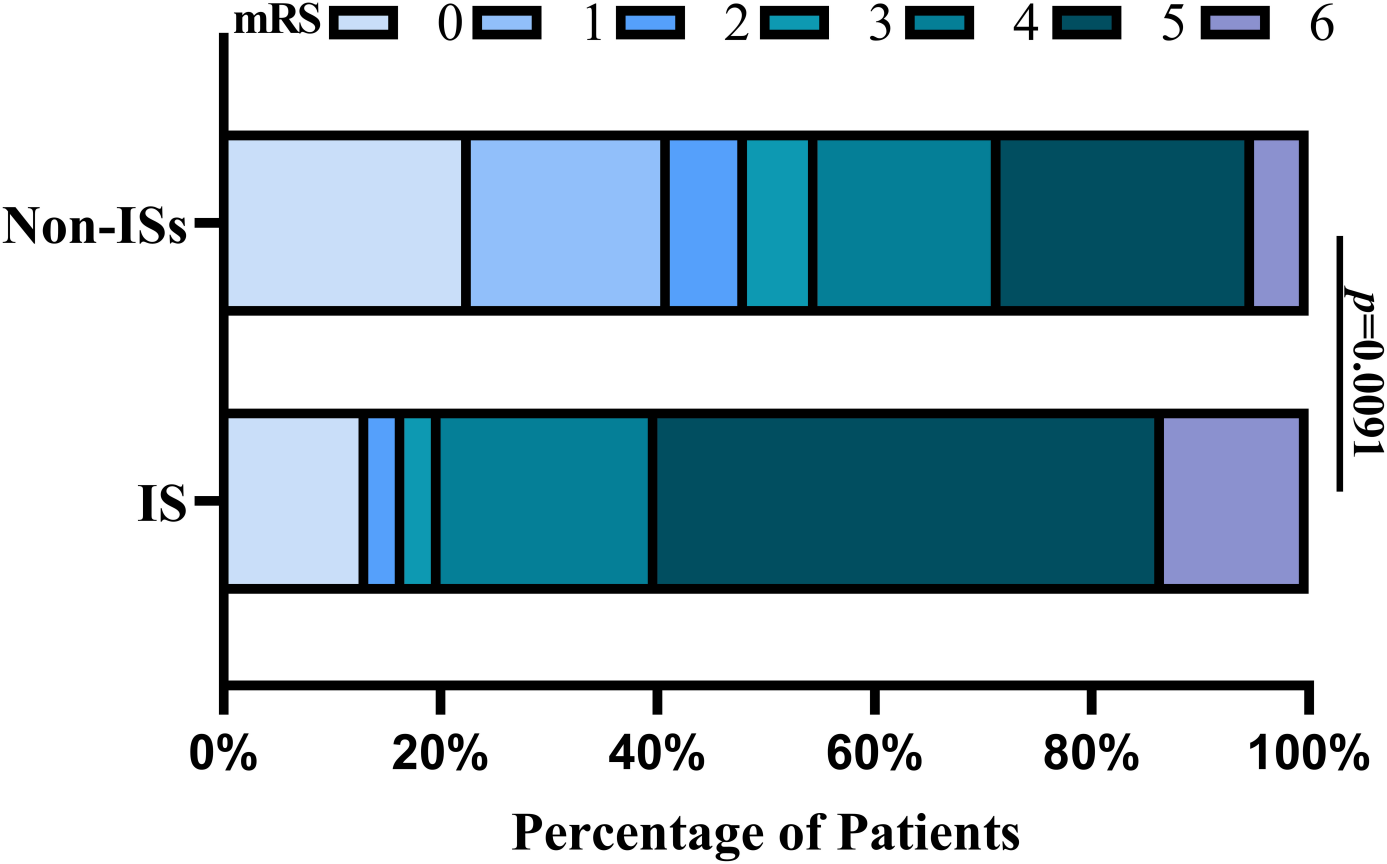
Distributions of mRS scores at 90 days between the ISs and non-ISS groups. Proportions of patients within each score category on the 7-point scale (where o indicates no symptoms and 6 indicates death) at 90 days. ISs, immediate seizures; mRS, modified Rankin Scale.

Patients with ISs had a statistically worse prognosis than patients without ISs (Table 1). For the functional outcome of mRS, the distribution of 90-day mRS of both groups is illustrated in Figure 4. Interestingly, no significant difference was witnessed between the two groups after PSM (Table 1).

No statistically significant difference was observed in the PaCO_2_ level between a favorable outcome and an unfavorable outcome [39.55(36.13–43.07) vs. 39.00(34.63–43.08) mmHg *p* = 0.341]. ROC analysis indicated an AUC of 0.529 for PaCO_2_ levels as a prognostic predictor in patients with ICH.

## Discussion

Four principal findings emerge from our study:(1) the lower initial PaCO_2_ (≤37.2 mmHg) level was a significant independent predictor of ISs in patients with primary ICH; (2) HE and younger age were significantly associated with ISs;(3) the initial PaCO_2_ level was positively associated with GCS score; and (4) patients with ISs had a statistically worse prognosis than patients without ISs, while no significant difference was witnessed between two groups after PSM. Following ICH, reported risk factors for ISs included age and HE (5, 12), which were balanced in this study. After PSM, there were no significant differences in age and HE between the two groups. The matched ISs group had lower initial PaCO_2_ levels compared with the matched non-ISs group in the univariate analysis. To the best of our knowledge, lower initial PaCO_2_ was first reported as a risk factor for ISs following ICH.

Acute hemorrhagic stroke was associated with a significant decrease in CO_2_ levels (10). Decreased PaCO_2_ can elevate the blood pH, which induces cerebral vasoconstriction in the brain and consequently reduces CBF and intracranial pressure (34, 35). Animal models frequently reported CO_2_ levels with epilepsy as a trigger for seizures (18, 19). Previous studies demonstrated that lower PaCO_2_ was associated with febrile convulsions or the absence seizure in children (23, 24). One recent study revealed that early decreased PaCO_2_ levels in the first 24 h were independently related to an increased incidence of acute seizures in patients receiving extracorporeal membrane oxygenation for respiratory failure (25). This study found that lower initial PaCO_2_ was a significant independent predictor of ISs following primary ICH, even after adjusting for confounders or PSM.

It has been well documented that CBF is associated with PaCO_2_ levels, and PaCO_2_ is one of the most decisive parameters that affect CBF (36). Middle cerebral artery (MCA) flow changes by 4% for every 1 mmHg increase or decrease in PaCO_2_ between 20 and 80 mmHg (16). Crucially, for every 1 mm Hg incremental lowering of PaCO_2_, CBF decreases by 3% (27). Prior study has hinted that an association between acute hemorrhagic stroke, lower PaCO_2_, and brain tissue hypoxia is purported to be driven by the onset of cerebral vasoconstriction (27, 37). Hextrum S and colleagues have reported that lower initial PaCO_2_ was associated with an increased risk of developing ischemic lesions (27). The vasoconstrictive effects of lower PaCO_2_ may be compounded by other factors that reduce CBF (acute blood pressure reduction in ICH, lowering intracranial pressure) to increase the risk of secondary ischemic injury (27), which results in the dysfunction of neuronal metabolic activity, thereby producing an acute epileptic seizure. Cerebral ischemia and reperfusion injury exacerbate seizures when CBF gradually returns to normal and subsequent oxygen and arterial PaCO_2_. In addition, the vasoconstrictive effects of lower PaCO_2_ reduce hemoglobin oxygen release, increasing neuronal excitability and possibly releasing excitatory toxins such as glutamate (38), which may induce ISs. Decreased PaCO_2_ can induce seizures by affecting cortical pH, γ-aminobutyric acid (GABA) release, and brain electrical activity (19). Lower cerebrospinal fluid (CSF) PaCO_2_ levels reduce cerebral blood volume and CBF through cerebral arterial vasoconstriction, which causes ischemia and hypoxia in brain tissue, contributing to ISs following ICH (39).

Clinical observations and experimental animal models have demonstrated that changes in CO_2_ have significant effects on neuronal excitability and seizure propensity in susceptible individuals (40–42). Reduced CO_2_ increases neuronal excitability in the hippocampus, contributing to acute seizures (40). The hippocampus, frequently involved in seizures, is susceptible to CO_2_ and mediates a neuroendocrine response to CO_2_ (40, 41). In the hippocampal slice preparation, decreasing CO_2_ levels reduced extracellular adenosine concentration and increased neuronal excitability via adenosine A_1_ receptors, adenosine triphosphate (ATP) receptors, and ecto ATPase, causing ISs (40).

As is well-known, inflammation response to ICH is a significant factor in acute seizures (26, 43). Shreds of evidence from clinical and experimental studies indicated that brain inflammation plays an essential role in seizures (26, 43). The elevation of inflammatory cytokines, such as tumor necrosis factor and interleukin, were also detected in patients with epilepsy and in epilepsy models (38, 43). Interestingly, lower PaCO_2_ can increase tumor necrosis factor and interleukin (38, 44). In the present study, decreased PaCO_2_ level was observed in the ISs group after ICH, supporting a possible link between PaCO_2_, inflammation, and seizures.

Interestingly, one unexpected finding is that the risk of ISs was no different between those with lobar and deep ICH, unlike the results of previous literature (12). The result is noteworthy for the following reasons. On the one hand, the classification of hematoma location in this study is different from previous literature (12). In a study by Cheng Qian et al. (12), which classified ICH locations into subcortical, thalamic, ganglionic, infratentorial (cerebellum and/or pons), and other hematomas, subcortical hematomas were associated with ISs. On the other hand, prior studies have focused on the relationship between early seizures (1–7 days or 1–14 days) and ICH location, rather than ISs (<24 h after admission) (15, 28). Early seizures and ISs have different temporal boundaries, and so the results are not universally identical.

The prognostic significance of PaCO_2_ levels in critically ill patients, including ICH, remains controversial. Hextrumet al. have previously demonstrated that lower initial PaCO_2_ was independently associated with a greater risk of in-hospital death in patients with ICH (27). A recent study has shown that decreased PaCO_2_ improves cerebral autoregulation and possible outcome in patients following ICH (45). Contrary to previous studies (25, 27, 45), we found that PaCO_2_ level was not associated with unfavorable outcomes. ROC analysis indicated an AUC of 0.529 for PaCO_2_ levels as a prognostic predictor in patients with ICH, indicating that the predictive value of PaCO_2_ level in predicting poor prognosis was weak. Lower initial PaCO_2_ level may be a marker of illness severity rather than a cause of worse clinical outcomes. This hypothesis supported the current study results of a positive correlation between the initial PaCO_2_ level and the commonly used disease severity scores-GCS score. The inconsistency of the data obtained in this study with previous studies may be due to the limited sample size and different inclusion and exclusion criteria.

The ISs incidence in our study is in line with the study of Szaflarski et al., demonstrating that 8.4% of patients with ICH had a seizure within the first 24 h of stroke onset in a population-based study (28). According to our definition of ISs, the incidence of ISs in the previous study is 8.4%. Whether there is an independent causal relationship between ISs and neurologic outcomes in patients with ICH remains controversial. Cheng Qian et al. have reported that ICH patients with ISs had the highest mortality rate (12). Conversely, Szaflarski JP et al. have demonstrated that ISs are not independent predictors of in-hospital mortality, which is consistent with the fact that ISs are predominantly lobar ICH without intraventricular spread and are smaller, implying a better prognosis (5). In the present study, patients with ISs had a statistically worse prognosis than patients without ISs at the 90-day follow-up after ICH onset.

This was a single-center clinical study and a hospital-based analysis with inherent limitations when interpreting the findings. Firstly, there were relatively few patients with ISs in the current research. Furthermore, PaCO_2_ levels of CSF had not been collected, and PaCO_2_ levels in arterial blood cannot directly reflect the actual level in the brain tissue. A single measurement may not fully reflect accurate PaCO_2_ levels over time. Continuous dynamic PaCO_2_ monitoring was the evident approach for improving accuracy. Thirdly, the sample sizes decreased, which may influence IS prediction accuracy for ISs due to the stringent inclusion/exclusion criteria. Fourthly, PaCO_2_ levels are within the normal range in some patients with ICH with ISs, so the result is not straightforward to interpret. More insight into detailed mechanisms awaits further study. Finally, the diagnosis of seizures was made clinically, and information on electroencephalography was not obtained.

## Conclusions

The lower initial PaCO_2_ levels may be a crucial biomarker in predicting ISs in patients following ICH. The PaCO_2_ level is readily available as an indicator of blood gas analysis at admission and may help identify high-risk patients with ISs. However, further large-scale or randomized studies are needed to verify these findings and determine whether changes in PaCO_2_ levels over time are associated with the onset of ISs.

## Data Availability Statement

The original contributions presented in the study are included in the article/supplementary material, further inquiries can be directed to the corresponding author/s.

## Ethics Statement

The studies involving human participants were reviewed and approved by the Research Ethics Committee of Dehua County Hospital. The patients/participants provided their written informed consent to participate in this study.

## Author Contributions

ZP and YZ designed the study. ZP, QZ, and YZ drafted the manuscript. ZP, CW, XC, and XL collected and analyzed data. YZ helped in the statistical analysis and result interpretation. XZ and YZ were identified as the guarantor of the article, taking responsibility for the integrity of the work as a whole, and prepared the figures and interpreted the results. JW, XZ, and YZ supervised the study and revised manuscript. All authors read and approved the final manuscript.

## Funding

This study was supported by the Science and Technology Planning Project of Quanzhou City (No. 2019NO11S to ZP) and Fujian University of Traditional Chinese Medicine School Management Project Clinical Special Funding (No. XB2021113 to CW).

## Conflict of Interest

The authors declare that the research was conducted in the absence of any commercial or financial relationships that could be construed as a potential conflict of interest.

## Publisher's Note

All claims expressed in this article are solely those of the authors and do not necessarily represent those of their affiliated organizations, or those of the publisher, the editors and the reviewers. Any product that may be evaluated in this article, or claim that may be made by its manufacturer, is not guaranteed or endorsed by the publisher.
